# Enteral *vs.* intravenous ICU sedation management: study protocol for a randomized controlled trial

**DOI:** 10.1186/1745-6215-14-92

**Published:** 2013-04-03

**Authors:** Giovanni Mistraletti, Elena S Mantovani, Paolo Cadringher, Barbara Cerri, Davide Corbella, Michele Umbrello, Stefania Anania, Elisa Andrighi, Serena Barello, Alessandra Di Carlo, Federica Martinetti, Paolo Formenti, Paolo Spanu, Gaetano Iapichino

**Affiliations:** 1 Dipartimento di Fisiopatologia medico-chirurgica e dei trapianti, Università degli Studi di Milano, A.O. San Paolo - Polo Universitario, Via A. Di Rudinì, Milan, 8-20142, Italy; 2 Dipartimento di Fisiopatologia medico-chirurgica e dei trapianti, Università degli Studi di Milano, I.R.C.C.S Ospedale Maggiore Policlinico, Milan, Italy; 3 Dipartimento di Anestesia e Rianimazione, Ospedali Riuniti di Bergamo, Bergamo, Italy

**Keywords:** Sedation, Hydroxyzine, Melatonin, Enteral approach, High-risk critically ill, Educational research

## Abstract

**Background:**

A relevant innovation about sedation of long-term Intensive Care Unit (ICU) patients is the ‘conscious target’: patients should be awake even during the critical phases of illness. Enteral sedative administration is nowadays unusual, even though the gastrointestinal tract works soon after ICU admission. The enteral approach cannot produce deep sedation; however, it is as adequate as the intravenous one, if the target is to keep patients awake and adapted to the environment, and has fewer side effects and lower costs.

**Methods/Design:**

A randomized, controlled, multicenter, single-blind trial comparing enteral and intravenous sedative treatments has been done in 12 Italian ICUs. The main objective was to achieve and maintain the desired sedation level: observed RASS = target RASS ± 1. Three hundred high-risk patients were planned to be randomly assigned to receive either intravenous propofol/midazolam or enteral melatonin/hydroxyzine/lorazepam. Group assignment occurred through online minimization process, in order to balance variables potentially influencing the outcomes (age, sex, SAPS II, type of admission, kidney failure, chronic obstructive pulmonary disease, sepsis) between groups. Once per shift, the staff recorded neurological monitoring using validated tools. Three flowcharts for pain, sedation, and delirium have been proposed; they have been designed to treat potentially correctable factors first, and, only once excluded, to administer neuroactive drugs. The study lasted from January 24 to December 31, 2012. A total of 348 patients have been randomized, through a centralized website, using a specific software expressly designed for this study. The created network of ICUs included a mix of both university and non-university hospitals, with different experience in managing enteral sedation. A dedicated free-access website was also created, in both Italian and English, for continuous education of ICU staff through CME courses.

**Discussion:**

This ‘educational research’ project aims both to compare two sedative strategies and to highlight the need for a profound cultural change, improving outcomes by keeping critically-ill patients awake.

**Trial registration number:**

Clinicaltrials.gov #NCT01360346

## Background

Agitation and anxiety are common in critically-ill patients [1]. They are triggered by treatable causes, such as hypoxemia, hypoglycemia, pain, sepsis, alcohol or drug withdrawal, invasive procedures, sleep deprivation, forced body postures, uninterrupted noise and light stimulation, and the impossibility of communicating with the staff [2]. International guidelines suggest to face and treat first any organic and/or metabolic cause, especially pain, and to minimize environment-linked stressors. Second, they suggest the use of sedatives to ensure comfort and permit life-saving procedures [2]: adequate levels of sedation, therefore, represent a primary target for managing Intensive Care Unit (ICU) patients. However, sedative therapy is related to several important side effects, among which are hemodynamic instability, dysrhythmias, sepsis, ileus, delirium, and lengthening of respiratory weaning [3].

Recently, several papers [4,5] have pointed out the need for lighter sedation, due both to the detrimental effects of sedative therapy itself and to the costs associated with deeper-than-needed sedation levels [6]. Daily interruption of intravenous short-term sedative administration is mandatory in neurosurgical or comatose patients. In a context of general ICU [7], Kress *et al*. demonstrated that, in association with a spontaneous breathing trial [8], it decreases mechanical ventilation length and reduces complications and prevalence of delirium. Analgesia-based sedation is gaining more and more popularity, due to the opiates’ ability to maintain adaptation to invasive procedures while sparing, at the same time, critically-ill patients’ consciousness [9]. However, the use of short-half-life analgesics (remifentanil) is only indicated for short-stay ICU patients, while there is no difference between opiates for patients with a length of stay (LOS) ≥ 3 days [10]. In 2010, Strom *et al*. demonstrated a clear indication to keep patients minimally sedated or awake by morphine boluses but without propofol [11].

The conscious sedation target is an innovation of the utmost relevance in the field of ICU care. Nonetheless, it has a number of detractors among intensivists, who tend to consider it unfeasible. Its unfeasibility is related to the potentially higher risk of self-removal of invasive devices [12] and stress/discomfort for patients [13]. From the ICU team prospective, it raises the possibility of an increased workload. These fears, however, are at least partially unfounded [14]: intensivists have to face side effects of both physical and ‘pharmacological’ restraint methods, continuously pursuing the best approach for patient security and healing.

**Figure 1:**
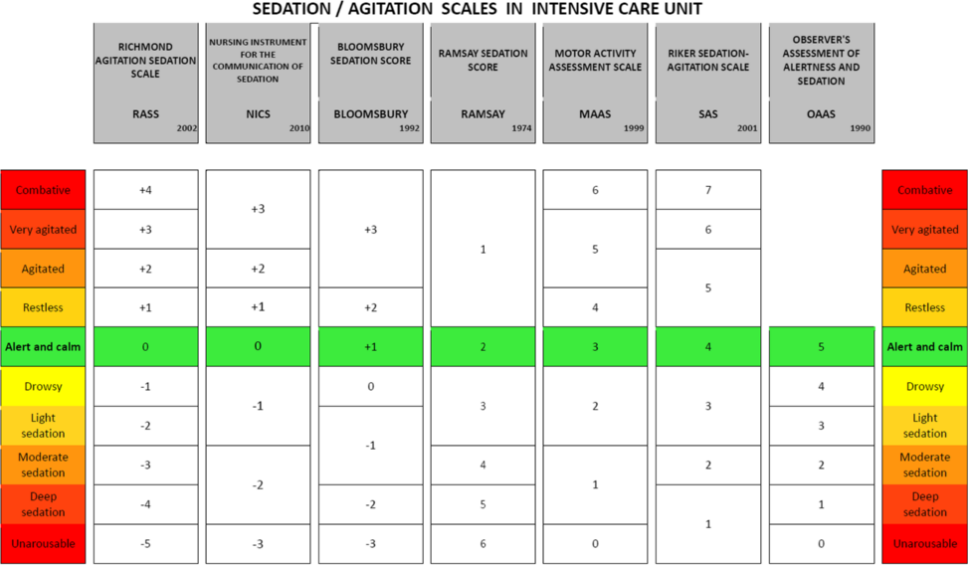
Comparison of most used ICU sedation/agitation scales highlighting the target of ‘conscious sedation’.

Despite guideline indications and the fact that between 60% and 80% of ICUs use a specific score to evaluate the level of sedation [2], many physicians habitually maintain [15,16] a deeper level of sedation than desired [17], probably causing avoidable side effects. The most commonly used score is Ramsay Sedation Scale (66.5%), although not validated, followed by Richmond Agitation Sedation Scale (RASS) (5.4%) [18], which, during the validation process, proved to be very reliable and to have low interpersonal variability both in the subgroups of the ventilated *vs.* not ventilated and the sedated *vs.* not sedated patients [19] (Figure 1). Beyond the choice of the specific drug, the most frequently used method for administering sedatives is the continuous intravenous route, because of its pharmacokinetic properties. Intravenous infusions present predictable and easy to handle onset/offset properties. Although these characteristics are necessary in short ICU stays, they can be unuseful, or even dangerous, for patients requiring > 3 days of mechanical ventilation [17]: when using potent drugs, it is easy to over-administer them, albeit goals are established and adequate [15]. Moreover, daily awakening trials produce far-from-physiological neurological fluctuations [20], and continuous deep sedation does not permit patients to recall factual memories, which has been proven effective in preventing post-traumatic stress disorder (PTSD) [21].

The enteral route for sedative administration is seldom used, even though the gastrointestinal tract is functional from the beginning of the ICU stay and in the most critical patients too [22,23]. Our group published two studies on the feasibility of enteral administration of hydroxyzine and lorazepam for ICU patient sedation [4,24]. Hydroxyzine, an antagonist of the H_1_ receptor, has anticholinergic, antiserotoninergic, antiemetic, and gastric antisecretory properties, and is frequently used in psychiatry and for pre-anesthetic sedation due to its neurological effects; also, since 2001, local hospital guidelines suggest the use of enteral hydroxyzine, with possible supplementation of lorazepam. Enteral melatonin is also administered as a physiological hypnoinducer (thus further saving on sedatives and analgesics): it does not cause excessive daytime sleepiness and presents interesting anti-oxidant, anti-inflammatory, and immune-modulating properties [25].

Enterally administered drugs are not as easy to manage as intravenous ones, due to their prolonged onset and offset. On the other hand, they provide a more stable level of consciousness, less cardiorespiratory depression, and are less expensive than intravenous drugs. They show an intrinsic impossibility to reach a deep sedation: exclusive enteral sedation has the same effectiveness (as judged by nurses) as intravenously administered drugs if a patient awake, tranquil, and well-adapted to the ICU environment is desired. Such a target can be reached in 83% of days following the first 48 h in ICU, after stabilization of clinical conditions [4,24].

The present study arises from this scientific framework; it aims to compare enteral *versus* commonly used intravenous sedative drug administration, measuring their efficacy and feasibility in reaching and constantly maintaining an appropriate sedation level (observed RASS = target RASS ± 1) [19] in high-risk critically-ill patients [26]. The clinical target is to obtain the most ‘conscious’ sedation level compatible with critical illnesses, invasive procedures, and medical and nursing surveillance. This paper describes the study protocol.

## Methods/Design

### Study design and outcomes

**Figure 2:**
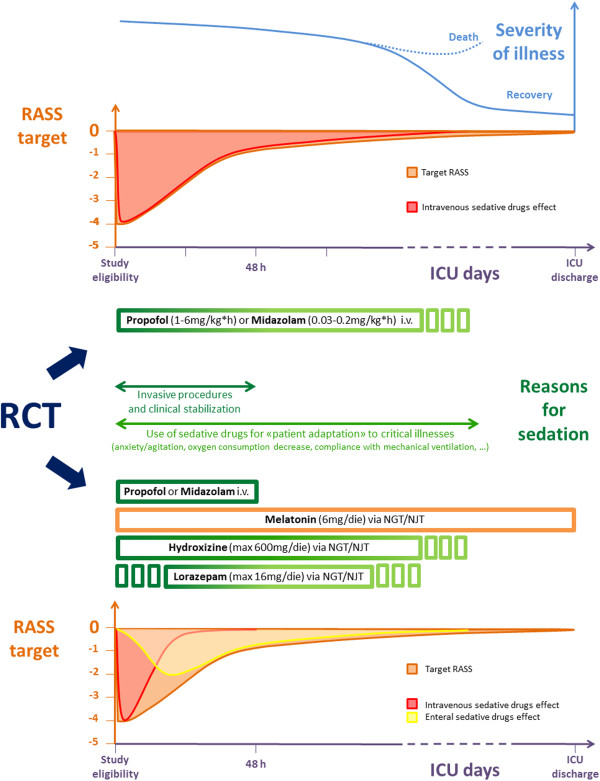
**Study protocol.** During the first 2 days of treatment, clinical conditions may require ‘deep sedation’ (RASS target from −4 to 0), achievable only by intravenous drugs. As soon as possible, even if critical conditions persist, the ‘conscious sedation’ period begins (RASS target −1/0). Within 24 h after meeting enrollment criteria, patients undergo a single-blind RCT lasting until ICU discharge: in the intravenous (control) arm, patients receive only i.v. drugs, according to ABC protocol [10]; in the enteral (intervention) arm, patients must interrupt i.v. sedatives within 48h, maintaining enteral ones until ICU discharge [25]. All sedatives have to be carefully tapered off to use the lowest effective dose. NGT, Naso-gastric tube; NJT, Naso-jejunal tube; RASS, Richmond Agitation Sedation Scale; RCT, Randomized controlled trial.

The Enteral *vs.* Intravenous Italian Trial (SedaEN) is a randomized, controlled, multicenter, single-blind trial (clinicaltrials.gov #NCT01360346), which aims to compare the efficacy of two different clinical approaches for sedative therapies (Figure 2): in the control group propofol and midazolam are continuously administered through intravenous route with daily interruption [8]. In the intervention group, sedation is maintained through enteral hydroxyzine, with possible supplementation of enteral lorazepam [24]; enteral melatonin will be administered as a physiological hypnoinducer [27]. Participating ICU centers were selected based on their willingness to simultaneously use two very different clinical approaches; the study group is heterogeneous, resulting in a mix of both university and non-university hospitals, with ICU teams having different experience in managing enteral sedation: go to Appendix to see all the nurses and physicians in charge at each participating ICU. This choice was made to obtain the best generalizability of the results.

The primary objective is to achieve and maintain the sedation target: observed RASS = target RASS ±1 [19]. Secondary outcomes are: sedation protocol feasibility (percentage of shifts with assigned protocol violations); delirium- and coma-free days, as assessed by Confusion Assessment Method for ICU (CAM-ICU) [28], and RASS [29]; ventilation-free days; nursing assessment of sedation adequacy (communication skills, cooperation, environment tolerance) [30]; length of ICU and hospital stay; ICU, hospital, and 1-year mortality; sedative drug costs; adverse events and markers of sedation failure, such as self-extubation, removal of other invasive tools, unscheduled diagnostic neurological tests, anxiety, hours of sleep and agitation, use of anti-psychotics, use of physical restraints, and pharmacological antagonists (Table 1).

**Table 1:** Primary and secondary study outcomes

***Primary outcome***	***Secondary outcomes***
Percentage of efficacy (measured as observed RASS = target RASS ±1)	- Sedation protocol effectiveness (percentage of ‘protocol violation days’ on the total of ICU days)
	- Delirium- and coma-free days (respectively negative CAM-ICU and RASS ≥ −3 in all daily observations until ICU day 28)
	- Ventilation-free days
	- Nursing evaluation of sedation adequacy (communication skills, cooperation, environment tolerance)
	- Overall ICU and hospital mortality
	- Absolute mortality 1 year after ICU discharge
	- Sedative drug costs
	- Indirect markers of inefficacy and side effects, like prevalence of ‘dangerous episodes’ (self-extubation, removal of other invasive tools), length of ICU and hospital stay, use of anti-psychotic drugs as indirect marker of delirium, indicators of sedation failure (use of physical restraints, antagonists administration like flumazenil or naloxone), sepsis prevalence.

### Study population

**Figure 3:**
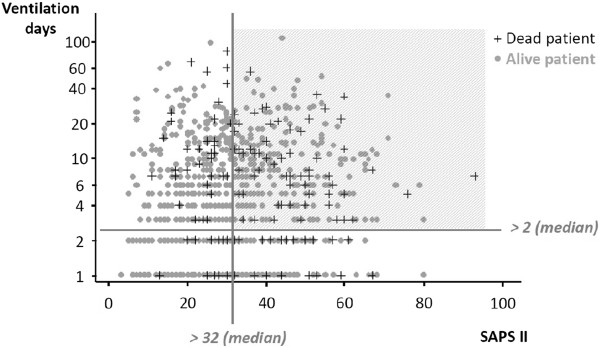
**Selection criteria for high-risk critically-ill patients.** Patients most representative of critical population present both high severity (SAPS higher than median) and high intensity (ventilation days higher than median) criteria. In these ICU patients, each therapeutic choice possibly influences clinical outcome [26].

All high-risk critically-ill patients (SAPS II ≥ 32, estimated length of mechanical ventilation ≥ 3 days) [26] (Figure 3) are eligible, within the first 24 h after they meet inclusion criteria. Figure 3 describes an ICU population of 7,877 critically-ill patients enrolled in an international, multicenter, observational trial: elderly in European intensive care units (ELDICUS). They had a mortality rate of 22.6% at 28 days and 29.6% at 90 days. This figure highlights the subgroup of patients with both severity and intensity of treatment higher than median values, whose clinical outcome is most likely to be highly influenced by therapeutic choices. Table 2 summarizes inclusion and exclusion criteria, including patients who are expected to need a kind or amount of sedatives different from usual care, because of their past or current medical history.

**Table 2:** Inclusion and exclusion criteria - patients are included if thay meet all three criteria, and are excluded if they meet one or more of the following criteria

***Inclusion criteria***	***Exclusion criteria***
SAPS II ≥ 32 points	Neurosurgical patients
Estimated length of mechanical ventilation at ICU admission ≥ 3 days	CNS diseases (epilepsy, stroke, dementia, post-anoxic coma)
Age ≥ 18 years	Liver encephalopathy (Child-Pugh C)
	Previous psychiatric or cognitive pathology
	Allergy to medications used in the study
	Absolute contraindications to the use of enteral route (nasogastric tube, nasojejunal tube, jejunostomy, ileostomy are all considered acceptable)
	Pregnant or breast-feeding patients
	Death is deemed imminent and inevitable or the patient has an underlying disease process with a life expectancy of < 90 days

### Study procedures

Both protocols are tailored to deliver the lowest effective doses of sedatives in order to reach as soon as possible and constantly maintain the target sedation level (RASS = 0) [14]. Moreover, ICU teams should try, whenever possible, to reduce or suspend all the sedative drugs. Physicians reconsider their target at every shift: if they choose a deep sedation goal (RASS < −3), their decision must be adequately justified. Even though every center is strongly advised not to make protocol violations, they are always feasible, provided they are explained and recorded. Intravenous boluses of fentanyl/morphine + propofol/midazolam are allowed in the enteral arm to perform and complete extemporary invasive/painful/surgical procedures; this will never represent a protocol violation.

**Figure 4:**
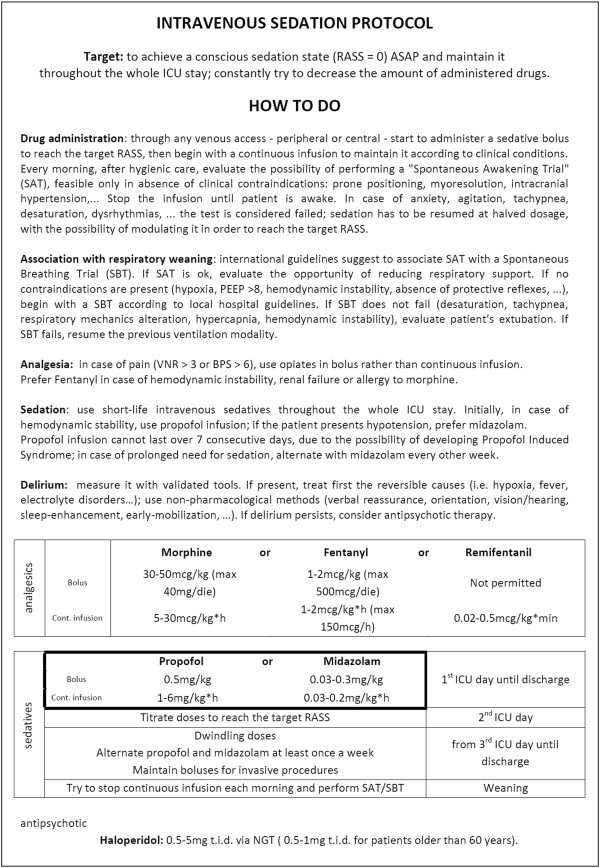
**Control arm: intravenous sedation, based on literature evidence[**[2]**,**[8]**].**

In the control arm of the study (‘intravenous’), propofol or midazolam (maximum: propofol 6 mg/kg*h; midazolam 0.2 mg/kg*h) are continuously administered intravenously from ICU admission until discharge, with daily interruption [8]. Enteral sedatives are always considered a protocol violation (Figure 4).

**Figure 5:**
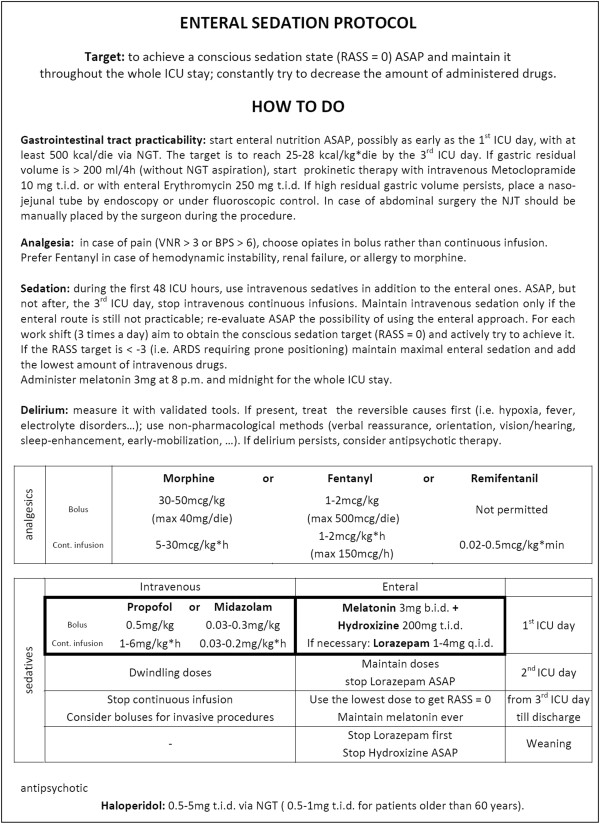
**Intervention arm: enteral sedation.** Local guidelines for conscious enteral sedation, adopted since 2001 in A.O. San Paolo - Polo Universitario, Milan, Italy following a multidisciplinary commission composed by intensivists, neurologists, and psychiatrists.

In the intervention arm (‘enteral’), intravenous sedatives are allowed during the first 48 h after ICU admission, in association with enteral sedatives. This is done to allow a more manageable sedative strategy during the first hours of ICU stay, when the highest number of procedures is performed and patients show the highest clinical instability. However, any continuous infusion must be stopped within 48 h. Sedation will be maintained through enteral hydroxyzine (maximum 600 mg/die) with the possible supplementation of lorazepam (maximum 16 mg/die). Enteral melatonin is administered from ICU admission until discharge, 3 mg b.i.d. (20:00 and midnight). Any intravenous sedative represents a protocol violation if target RASS ≥ −3. In case of clinical indication for deeper sedation, once enteral drugs are maximized, the necessary intravenous administration is not considered a protocol violation (Figure 5).

**Figure 6:**
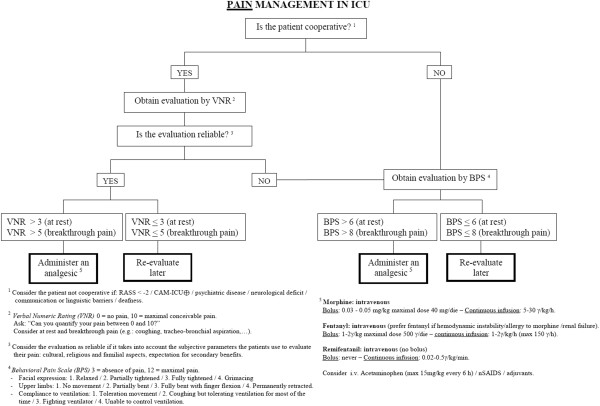
Bedside flowchart for pain management in ICU patients.

**Figure 7:**
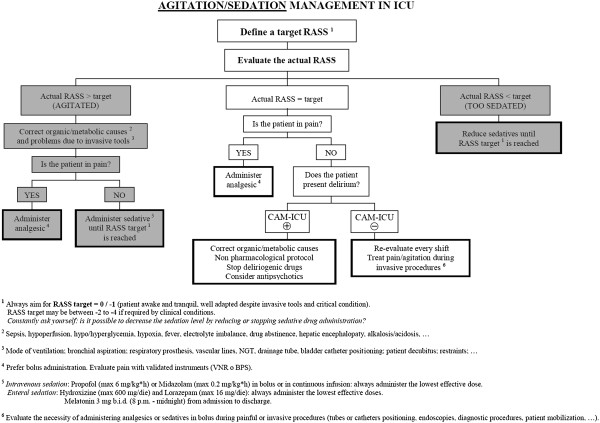
Bedside flowchart for sedation/agitation management in ICU patients.

**Figure 8:**
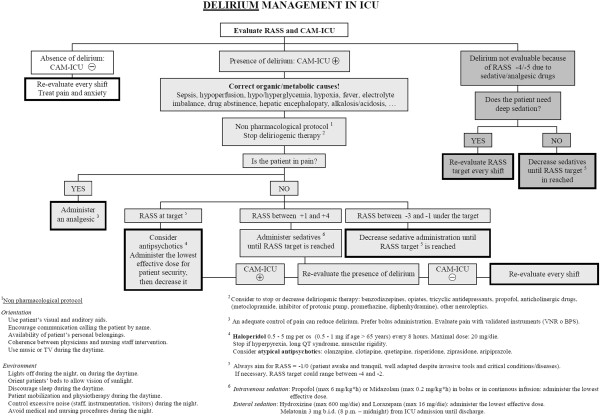
Bedside flowchart for delirium management in ICU patients.

Analgesia, delirium, and respiratory weaning will be managed in accordance with local guidelines; to help manage particularly complex cases, three flowcharts are proposed, for pain, sedation, and delirium (Figures 6, 7 and 8). They stress the importance of considering all those factors (that is, organic and metabolic causes, presence of invasive tools, pain, and so on) that could contribute to agitation and anxiety, and should be corrected before administering any neuroactive drug. Participating centers must use analgesics (particularly opiates) only in case of pain (Verbal Numeric Rating (VNR) > 3; or Behavioral Pain Scale (BPS) > 6), [31] avoiding analgesia-based sedation. If delirium appears (CAM-ICU +), after correcting underlying causes, the non-pharmacological protocol will be applied and potential deliriogenic therapies stopped; only after these interventions may physicians prescribe haloperidol (1 mg per os, maximum 10 mg/die), or any other antipsychotic drug, according to local guidelines.

**Figure 9:**
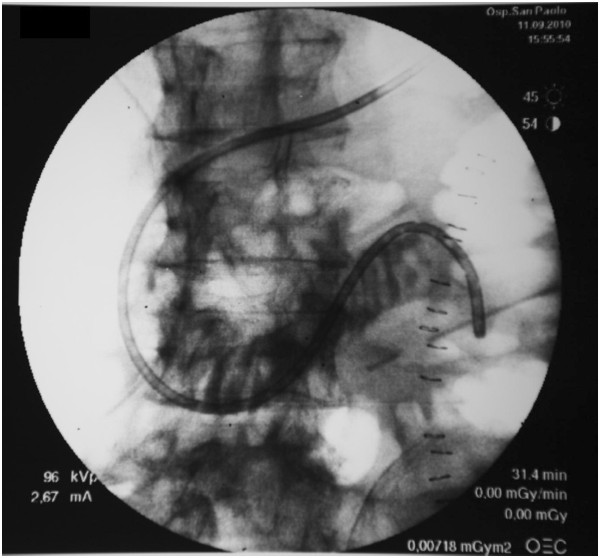
Post-pyloric access: guide-wired nasojejunal tube positioned under fluoroscopy.

Both groups will start enteral nutrition during the first 24 to 48 ICU hours, while parenteral nutrition will be administered only to achieve the caloric target if not reached by enteral route [32]. In case of gastric residual volume > 200 mL/4h, prokinetic drugs will be used according to local guidelines; if the problem persists for > 2 days, a post-pyloric access is recommended: auto-propulsive naso-jejunal tube (NJT), or guide-wired NJT positioned under fluoroscopy (Figure 9), endoscopic-positioned NJT, post-pyloric tube manually positioned by the surgeon, or percutaneous jejunostomy during open-abdomen operations.

All patients are maintained in sitting semi-orthopnoeic position (headboard of the bed elevated between 30° and 45°). Physiotherapy is started as soon as possible, according to local guidelines.

### Randomization process

**Figure 10:**
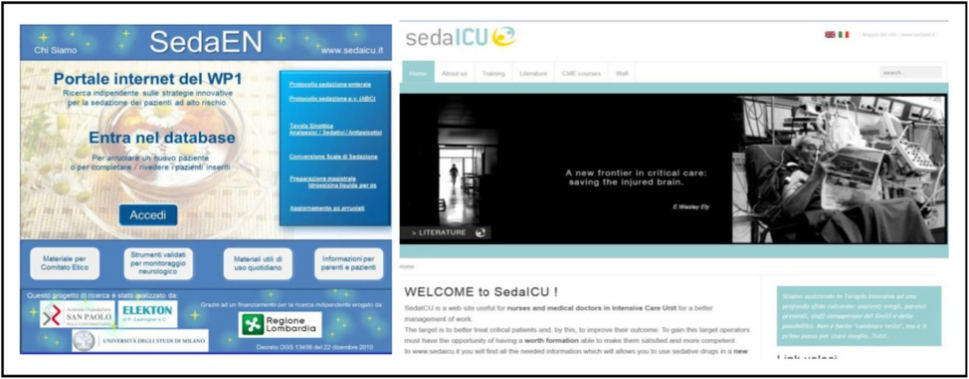
**Home pages of the two Internet websites specifically designed for the SedaEN study.**http://www.sedaen.it is the website where patients are randomized and patients’ data are recorded, while http://www.sedaicu.it/en is available to all nurses and physicians 24/7, containing materials for the correct use of validated tools for neurological monitoring of critically-ill patients, both in Italian and English.

Patient allocation to the two treatment groups is made by an online process over a password-protected, interactive, centralized website, through specific software, available 24/7, expressly designed for this study (http://www.sedaen.it (Figure 10)). After checking for inclusion and exclusion criteria, trained staff at each participating center obtain informed consent according to local Ethical Committee indications. Patient allocation occurs using a minimization algorithm [33], to maintain groups balanced within each center, according to those characteristics potentially influencing the study outcomes, as indicated by the SedaEN Steering Committee. Each variable’s weight is: Simplified Acute Physiology Score (SAPS II) [34], 10; type of admission, 6; chronic or acute kidney failure, 5; severe sepsis or septic shock, 4; COPD, 3; age, 2; sex, 1. Using this method, the first patient enrolled in each center is randomly assigned to one of the study arms by a computer-generated order. This allocation is the basis for all subsequent assignments for that center, unless there is perfect balance among admission characteristics; in this case, the next patient is once again randomly allocated. A full explanation of the minimization process is available at http://www.elekton.it/randomind. Once a patient is assigned, due to the intention-to-treat design of the study, the change of protocol arm is not possible for any reason.

### Data collection and management

Trained staff (see Appendix) record data and fill in the web-based collection forms. Before group assignment, baseline patient demographics and characteristics for minimization are collected. From the day after patient allocation, information on daily physiological parameters are used to derive SOFA score [35] and level of care [36]. Three times a day, once per shift, in addition to vital and lab parameters, neurological monitoring through validated tools is performed and recorded. In particular, target RASS (established by the physician in charge) and the actual RASS, [19] that is the ‘prevalent level’ in the shift, VNR for anxiety and pain (BPS if patient is uncooperative) [31], CAM-ICU [28], and nursing evaluation on adequacy of prescribed sedative therapy is recorded. Protocol violations and their clinical motivation, along with doses of analgesics, sedatives, and antipsychotics used for ‘rescue therapy’, are also recorded.

At ICU discharge, patient data and vital status, length of mechanical ventilation, and ICU stay are recorded. At hospital discharge, length of stay and mortality are registered. One year after ICU discharge, mortality will be assessed through a phone interview.

The web-based data management system allows for *ad hoc* and automatic validation and consistency checks as well as immediate query resolution. This ensures data accuracy and completeness; it allows timely access to ‘clean’ data for analysis purposes. Finally, a screening log will be maintained at each participating center to record ICU admitted patients who were considered ineligible.

### Ethical issues

All participating centers obtained local ethics committee approvals to conduct the trial: ‘Comitato Etico’ of A.O. San Paolo - Polo Universitario, Milano; ‘Comitato Etico Interaziendale’ for the centers A.O.N. SS. Antonio e Biagio e Cesare Arrigo, Alessandria, and A.O. Ospedale Cardinal Massaia, Asti; ‘Comitato Etico’ of A. O. Ospedale Civile di Desio (MI); ‘Comitato Etico’ of A.O. Ospedale Civile di Legnano (MI); ‘Comitato etico’ of Nuovo Ospedale Civile Sant’Agostino Estense, Modena; ‘Comitato Etico’ of A.O. San Gerardo, Monza (MB); ‘Comitato Etico’ of A.O.U. San Luigi Gonzaga, Orbassano (TO); ‘Comitato Etico’ of I.R.C.C.S Ospedale Maggiore Policlinico, Milano; ‘Comitato Etico’ of I.R.C.C.S. San Matteo, Pavia; and ‘Comitato Etico’ of A.O. San Giovanni Bosco, Torino. Written informed consent is mandatory for all able patients. In cases of impossibility to obtain it, a written declaration of received information is collected from relatives, according to local ethics committee indications. As soon as neurological conditions improve, patients are duly informed of the study, and their written consent is obtained. Patients or their next of kin have the opportunity to withdraw from the study at any time.

### Sample size and power

A study sample size of 300 patients was calculated in order to observe a 15% difference in the prevalence of sedation adequacy (observed RASS = target RASS ±1) between the two study arms: such a difference is considered clinically relevant and likely to influence practice. Considering that the enteral approach allowed for 83% adequacy in an observational monocentric study [24], it was considered necessary to enroll 141 patient per arm (power 80%, alpha 0.05). Considering missing data, 300 patients were expected to be enrolled among the 12 centers. Each center had to enroll at least 20 patients during the study period, otherwise it would not be considered for the planned sub-analysis comparing the participating ICUs.

### Statistical analysis

Statistical analysis will be conducted on an intention-to-treat basis, due to the potential high violation rate from the assigned protocol. A ‘per-protocol’ planned analysis will be conducted, excluding patients receiving no analgesic or sedative drugs, having an ICU stay < 48 h, or presenting assigned protocol violations in every ICU day. Subgroup analyses are planned *a priori* to obtain a comparison: (1) among participating ICUs, because of dissimilar ability to manage the two different sedative protocols; (2) between septic and non-septic patients (greater delirium prevalence, lower levels of endogenous melatonin) [37]; and (3) between different age groups (greater delirium prevalence if age > 70 years, higher risk of paradoxical effect of benzodiazepines, impairment of renal and hepatic function) [38].

An ‘*ad interim*’ analysis was planned after the enrollment of 70 patients per group. Its purpose was to identify any potential safety issues or test for early efficacy. It was evaluated by the SedaEN Steering Committee (see Appendix): since no safety issues were reported, and interesting trends were found about the main outcome, patients’ enrollment was completed according to the established planning.

Baseline patient characteristics and single-observation outcomes will be analyzed by Student *T*-test for continuous measures if normally distributed; by Wilcoxon rank-sum test if not normally distributed; by Fisher’s exact test for categorical measures. Appropriate (linear, logistic, or Poisson) regression models will be generated for the identification of the determinants of outcomes and for the correction for baseline covariates.

Mortality will be compared using relative risks and 95% confidence intervals. Survival times will be compared by means of the log-rank test and presented as Kaplan-Meier curves without adjustment for baseline covariates and as multivariate Cox proportional hazard analysis.

Analysis for repeated measures will be performed for data recorded during the whole ICU stay, that is, the main outcome: comparisons will be made by cross-sectional time-series regression models (random-effects, and population-averaged linear models) or by multilevel mixed-effects Poisson regressions, when appropriate [39]. Sepsis prevalence during the whole ICU stay will be analyzed by conditional fixed-effects logistic regression. This statistical approach was chosen to simultaneously analyze the net effect of group assignment; the effect of time spent in ICU; the cumulative sedative effect, calculated by multiplying the group (enteral = 1, intravenous = 0) and the ICU day from group assignment, to highlight the adjunctive effects of the daily repeated sedative administration.

For the *a priori* planned sub-analyses, outcomes and methods will be the same as for the main analysis. Missing data regarding the main outcome should be absent or very few, since the centralized website needed the completeness of data in order to permit the final validation of each patient’s form. For the other data, missing observations will be handled with multiple imputations as calculated by the statistical package Stata 12 (Stata Corporation, College Station, TX, USA). This software will be used for all the statistical analyses.

## Discussion

This ‘educational research’ project aims both to compare two sedative strategies and to highlight the need for a profound cultural change, improving outcomes by keeping critically-ill patients awake.

### Study limitations

This study has some limitations: first, it is a single-blind study, since the two different routes to give sedatives do not permit staff blindness to drug administration. To cope with this problem, great efforts in order to maintain the same sedation target in both study arms were made: many ready-to-use tools were provided to nurses and physicians; an oral presentation was made by the principal investigator in all participating centers; two investigators’ meetings (on March 23 and September 15, 2012) were organized. Second, two relevant essentials of the study (conscious target and use of validated tools for neurological monitoring) were introduced in some of the participating centers together with the beginning of the study; in others, they were already consolidated in clinical practice. Since one of the goals is the promotion of a new culture of sedation management, the simultaneous presence of different skills among intensivist staff makes the results more generalizable, even if they come from non-homogeneous ICUs.

### Training for ICU staff: the new concept of educational research

Although conscious sedation is now recognized as valid by solid scientific evidence, international literature widely describes cultural and organizational difficulties in introducing it into clinical practice, and in using daily validated tools for neurological monitoring of ICU patients [14].

Our study aims to be part of an ‘educational research’ project: the comparison of two different protocols, within the framework of a common target (conscious sedation) represents some kind of innovation, thus fulfilling a scientific goal, since at the present time both these protocols are described in the Italian guidelines [40]. Beyond scientific questions, the introduction of validated monitoring instruments into clinical practice, required for data collection, moves closer to international indications. Through teaching and using validated tools, ICU operators become aware of the need for a cultural change, in order to improve critically-ill patient care by keeping them awake. In this context, an important part of the present trial is to offer training materials, available 24/7 for everyday use, through a dedicated free-access website (http://www.sedaicu.it/en), both in Italian and English, which offers continuous education to physicians and nurses through CME courses.

## Trial status

**Figure 11:**
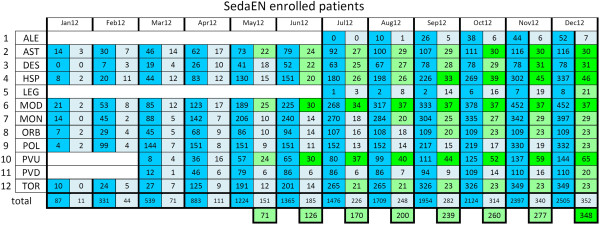
**Number of patients admitted to the SedaEN participating ICUs from January 24 to December 31, 2012.** In blue: all patients recorded on the website. In light blue: patients randomized. In light green: patients from centers included in the planned sub-analysis comparing the participating ICUs (at least 20 per center). In green: patients from centers meeting the requested number of enrolments (30 patients). ALE, A.O.N. SS. Antonio e Biagio e Cesare Arrigo, Alessandria; AST, A.O. Ospedale Cardinal Massaia, Asti; DES, A. O. Ospedale Civile di Desio (MI); HSP, A.O. San Paolo - Polo Universitario, Milano; LEG, A.O. Ospedale Civile di Legnano (MI); MOD, Nuovo Ospedale Civile Sant’Agostino Estense, Modena; MON, A.O. San Gerardo, Monza (MB); ORB, A.O.U. San Luigi Gonzaga, Orbassano (TO); POL, I.R.C.C.S. Ospedale Maggiore Policlinico, Milano; PVU e PVD, I.R.C.C.S. San Matteo, Università degli Studi di Pavia; TOR, A.O. San Giovanni Bosco, Torino.

Patients’ enrollment lasted from January 24 to December 31, 2012. A total of 348 patients have been randomized, through a centralized website, using the specific software expressly designed for this study. Figure 11 shows the enrollment course across the participating centers.

## Appendix

### Steering Committee members

Gaetano Iapichino (Study Chair, Dipartimento di Fisiopatologia medico-chirurgica e dei trapianti, Università degli Studi di Milano, A.O. San Paolo, Milano), Alberto Morabito (Chair of Statistics, Dipartimento di Scienze cliniche e di comunità, Università degli Studi di Milano, A.O. San Paolo, Milano), Martin Langer (Dipartimento di Fisiopatologia medico-chirurgica e dei trapianti, Università degli Studi di Milano, Fondazione I.R.C.C.S. - Istituto Nazionale dei Tumori, Milano), Roberto Malacrida (Cure Intense, Ospedale Regionale di Lugano, Switzerland), Franco Valenza (Dipartimento di Fisiopatologia medico-chirurgica e dei trapianti, Università degli Studi di Milano, I.R.C.C.S. Ospedale Maggiore Policlinico, Milano), Marco Rambaldi (Nuovo Ospedale Civile Sant’Agostino Estense, Modena), Giovanni Mistraletti (Dipartimento di Fisiopatologia medico-chirurgica e dei trapianti, Università degli Studi di Milano, A.O. San Paolo, Milano).

### Participating centers and SedaEN investigators

A.O. San Paolo - Polo Universitario, Milano: Andrea Noto, Gianfranco Cappello, Bruno Sabatelli, Giovanni Brenna, Morena Astori, Cristina Villa, and Pietro Placido.

I.R.C.C.S Ospedale Maggiore Policlinico, Milano: Luciano Gattinoni, Alessandro Protti, Riccarda Russo, Francesca Pagan, Virna Berto, Cristina Carin Sparacino, and Paola Roselli.

A. O. Ospedale Civile di Desio (MI): Giulio Ronzoni, Silvia Francesconi, Eduardo Beck, and Maurizio Gaiotto.

A.O. Ospedale Civile di Legnano (MI): Danilo Radrizzani, Luca Ferla, Federico Valdambrini, Riccardo Giudici, and Laura Merlini.

A.O. San Gerardo, Monza (MB): Antonio Pesenti, Giacomo Bellani, Emanuele Rezoagli, Alessia La Bruna, and Alberto Lucchini.

I.R.C.C.S. San Matteo, Università degli Studi di Pavia: AR1 Antonio Braschi, Alessandra Palo, Thekla Niebel, Marina Selvini, Sergio Cortesi and Attilio Quaini; AR2 Giorgio Iotti, Francesca Riccardi, and Antonella Sacchi.

A.O. San Giovanni Bosco, Torino: Sergio Livigni, Enrica Ferretti, Giuseppe Naretto, Alessandro Deprado, Tiziana Casalicchio, and Virna Venturi degli Esposti.

A.O.U. San Luigi Gonzaga, Orbassano (TO): Giulio Radeschi, Maurilio Festa, Lorenzo Odetto, Daniele Ferrero, Stefano Cognolato, Roberto Penso, and Roberta Vacchelli.

A.O. Ospedale Cardinal Massaia, Asti: Silvano Cardellino, Edda Bosco, Anna Maria Gado, Anna Bresciani, Ivana Pozzo, Annachiara Alessio, Vanessa Clarindo Rodrigues, and Edna Biase.

A.O.N. SS. Antonio e Biagio e Cesare Arrigo, Alessandria: Nicoletta Vivaldi, Antonella Nava.

Nuovo Ospedale Civile Sant’Agostino Estense, Modena: Marco Rambaldi, Cristina Pinna, Francesco Ponzetta, Lucilla Bavutti, Paola Martina, Beatriz Palacios, and Giancarla Bergonzini.

## Competing interests

All the authors, the steering committee members, and the SedaEN investigators declare they have no competing interests.

## Authors’ contributions

GM is the principal investigator of the study and responsible for the conception, protocol design, and organization of the financial support. GI submitted and received the financial support by Regione Lombardia to initiate this clinical trial. MU has provided statistical guidance, is responsible for the estimation of the sampling size and final statistical analysis. PC is responsible for designing and managing the specific software used for patient allocation in this study. GM, SA, SB, EA, AD, and FM are involved in the management of the study and responsible for data acquisition and study coordination. ESM, BC, and GM wrote the manuscript; GI, DC, PF, and PS revised the draft for important intellectual content. All authors have read and approved the final version and submission of the present manuscript to *Trials*.

## References

[B1] Fraser GL Riker RR Sedation and analgesia in the critically ill adult Curr Opin Anaesthesiol 2007 20 119 123 10.1097/ACO.0b013e32808255b4 17413394

[B2] Martin J Heymann A Basell K Baron R Biniek R Burkle H Dall P Dictus C Eggers V Eichler I Engelmann L Garten L Hartl W Haase U Huth R Kessler P Kleinschmidt S Koppert W Kretz FJ Laubenthal H Marggraf G Meiser A Neugebauer E Neuhaus U Putensen C Quintel M Reske A Roth B Scholz J Schroder S Evidence and consensus-based German guidelines for the management of analgesia, sedation and delirium in intensive care–short version Ger Med Sci 2010 8 Doc02 20200655 10.3205/000091 PMC2830566

[B3] Riker RR Fraser GL Adverse events associated with sedatives, analgesics, and other drugs that provide patient comfort in the intensive care unit Pharmacotherapy 2005 25 8S 18S 10.1592/phco.2005.25.5_Part_2.8S 15899744

[B4] Cigada M Corbella D Mistraletti G Forster CR Tommasino C Morabito A Iapichino G Conscious sedation in the critically ill ventilated patient J Crit Care 2008 23 349 353 10.1016/j.jcrc.2007.04.003 18725039

[B5] De Jonghe B Bastuji-Garin S Fangio P Lacherade JC Jabot J Appere-De-Vecchi C Rocha N Outin H Sedation algorithm in critically ill patients without acute brain injury Crit Care Med 2005 33 120 127 10.1097/01.CCM.0000150268.04228.68 15644658

[B6] Sessler CN Varney K Patient-focused sedation and analgesia in the ICU Chest 2008 133 552 565 10.1378/chest.07-2026 18252923

[B7] Kress JP Pohlman AS O’Connor MF Hall JB Daily interruption of sedative infusions in critically ill patients undergoing mechanical ventilation N Engl J Med 2000 342 1471 1477 10.1056/NEJM200005183422002 10816184

[B8] Girard TD Kress JP Fuchs BD Thomason JW Schweickert WD Pun BT Taichman DB Dunn JG Pohlman AS Kinniry PA Jackson JC Canonico AE Light RW Shintani AK Thompson JL Gordon SM Hall JB Dittus RS Bernard GR Ely EW Efficacy and safety of a paired sedation and ventilator weaning protocol for mechanically ventilated patients in intensive care (Awakening and Breathing Controlled trial): a randomised controlled trial Lancet 2008 371 126 134 10.1016/S0140-6736(08)60105-1 18191684

[B9] Breen D Karabinis A Malbrain M Morais R Albrecht S Jarnvig IL Parkinson P Kirkham AJ Decreased duration of mechanical ventilation when comparing analgesia-based sedation using remifentanil with standard hypnotic-based sedation for up to 10 days in intensive care unit patients: a randomised trial [ISRCTN47583497] Crit Care 2005 9 R200 R210 10.1186/cc3495 15987391 PMC1175879

[B10] Spies C Macguill M Heymann A Ganea C Krahne D Assman A Kosiek HR Scholtz K Wernecke KD Martin J A prospective, randomized, double-blind, multicenter study comparing remifentanil with fentanyl in mechanically ventilated patients Intensive Care Med 2011 37 469 476 10.1007/s00134-010-2100-5 21165734

[B11] Strom T Martinussen T Toft P A protocol of no sedation for critically ill patients receiving mechanical ventilation: a randomised trial Lancet 2010 375 475 480 10.1016/S0140-6736(09)62072-9 20116842

[B12] Chevron V Menard JF Richard JC Girault C Leroy J Bonmarchand G Unplanned extubation: risk factors of development and predictive criteria for reintubation Crit Care Med 1998 26 1049 1053 10.1097/00003246-199806000-00026 9635654

[B13] Guttormson JL Chlan L Weinert C Savik K Factors influencing nurse sedation practices with mechanically ventilated patients: a U.S. national survey Intensive Crit Care Nurs 2010 26 44 50 10.1016/j.iccn.2009.10.004 19945879

[B14] Goodwin H Lewin JJ Mirski MA ‘Cooperative sedation’: optimizing comfort while maximizing systemic and neurological function Crit Care 2012 16 217 10.1186/cc11231 22429840 PMC3681362

[B15] Martin J Franck M Fischer M Spies C Sedation and analgesia in German intensive care units: how is it done in reality? Results of a patient-based survey of analgesia and sedation Intensive Care Med 2006 32 1137 1142 10.1007/s00134-006-0214-6 16741692

[B16] Payen JF Chanques G Mantz J Hercule C Auriant I Leguillou JL Binhas M Genty C Rolland C Bosson JL Current practices in sedation and analgesia for mechanically ventilated critically ill patients: a prospective multicenter patient-based study Anesthesiology 2007 106 687 695 quiz, 891–892 10.1097/01.anes.0000264747.09017.da 17413906

[B17] Devlin JW The pharmacology of oversedation in mechanically ventilated adults Curr Opin Crit Care 2008 14 403 407 10.1097/MCC.0b013e32830280b3 18614903

[B18] Reschreiter H Maiden M Kapila A Sedation practice in the intensive care unit: a UK national survey Crit Care 2008 12 R152 10.1186/cc7141 19046459 PMC2646317

[B19] Sessler CN Gosnell MS Grap MJ Brophy GM O’Neal PV Keane KA Tesoro EP Elswick RK The Richmond Agitation-Sedation Scale: validity and reliability in adult intensive care unit patients Am J Respir Crit Care Med 2002 166 1338 1344 10.1164/rccm.2107138 12421743

[B20] Schweickert WD Kress JP Strategies to optimize analgesia and sedation Crit Care 2008 Suppl 3 S6 18495057 10.1186/cc6151 PMC2391265

[B21] Jones C Griffiths RD Humphris G Skirrow PM Memory, delusions, and the development of acute posttraumatic stress disorder-related symptoms after intensive care Crit Care Med 2001 29 573 580 10.1097/00003246-200103000-00019 11373423

[B22] Mistraletti G Sabbatini G Taverna M Figini MA Umbrello M Magni P Ruscica M Dozio E Esposti R DeMartini G Fraschini F Rezzani R Reiter RJ Iapichino G Pharmacokinetics of orally administered melatonin in critically ill patients J Pineal Res 2010 48 142 147 10.1111/j.1600-079X.2009.00737.x 20070489

[B23] Iapichino G Pesenti A Radrizzani D Solca M Pelizzola A Gattinoni L Nutritional support to long-term anesthetized and curarized patients under extracorporeal respiratory assist for terminal pulmonary failure JPEN J Parenter Enteral Nutr 1983 7 50 54 10.1177/014860718300700150 6403732

[B24] Cigada M Pezzi A Di Mauro P Marzorati S Noto A Valdambrini F Zaniboni M Astori M Iapichino G Sedation in the critically ill ventilated patient: possible role of enteral drugs Intensive Care Med 2005 31 482 486 10.1007/s00134-005-2559-7 15714324

[B25] Bellapart J Boots R Potential use of melatonin in sleep and delirium in the critically ill Br J Anaesth 2012 108 572 580 10.1093/bja/aes035 22419624

[B26] Iapichino G Mistraletti G Corbella D Bassi G Borotto E Miranda DR Morabito A Scoring system for the selection of high-risk patients in the intensive care unit Crit Care Med 2006 34 1039 1043 10.1097/01.CCM.0000206286.19444.40 16484895

[B27] Bourne RS Mills GH Minelli C Melatonin therapy to improve nocturnal sleep in critically ill patients: encouraging results from a small randomised controlled trial Crit Care 2008 12 R52 10.1186/cc6871 18423009 PMC2447606

[B28] Ely EW Margolin R Francis J May L Truman B Dittus R Speroff T Gautam S Bernard GR Inouye SK Evaluation of delirium in critically ill patients: validation of the Confusion Assessment Method for the Intensive Care Unit (CAM-ICU) Crit Care Med 2001 29 1370 1379 10.1097/00003246-200107000-00012 11445689

[B29] Pandharipande PP Pun BT Herr DL Maze M Girard TD Miller RR Shintani AK Thompson JL Jackson JC Deppen SA Stiles RA Dittus RS Bernard GR Ely EW Effect of sedation with dexmedetomidine vs lorazepam on acute brain dysfunction in mechanically ventilated patients: the MENDS randomized controlled trial JAMA 2007 298 2644 2653 10.1001/jama.298.22.2644 18073360

[B30] Riker RR Shehabi Y Bokesch PM Ceraso D Wisemandle W Koura F Whitten P Margolis BD Byrne DW Ely EW Rocha MG Dexmedetomidine vs midazolam for sedation of critically ill patients: a randomized trial JAMA 2009 301 489 499 10.1001/jama.2009.56 19188334

[B31] Ahlers SJ van der Veen AM van Dijk M Tibboel D Knibbe CA The use of the Behavioral Pain Scale to assess pain in conscious sedated patients Anesth Analg 2010 110 127 133 10.1213/ANE.0b013e3181c3119e 19897804

[B32] Kreymann KG Berger MM Deutz NE Hiesmayr M Jolliet P Kazandjiev G Nitenberg G van den Berghe G Wernerman J Ebner C Hartl W Heymann C Spies C ESPEN Guidelines on Enteral Nutrition: Intensive care Clin Nutr 2006 25 210 223 10.1016/j.clnu.2006.01.021 16697087

[B33] Altman DG Bland JM Treatment allocation by minimisation BMJ 2005 330 843 10.1136/bmj.330.7495.843 15817555 PMC556084

[B34] Le Gall JR Lemeshow S Saulnier F A new Simplified Acute Physiology Score (SAPS II) based on a European/North American multicenter study JAMA 1993 270 2957 2963 10.1001/jama.1993.03510240069035 8254858

[B35] Vincent JL Moreno R Takala J Willatts S De Mendonca A Bruining H Reinhart CK Suter PM Thijs LG The SOFA (Sepsis-related Organ Failure Assessment) score to describe organ dysfunction/failure. On behalf of the Working Group on Sepsis-Related Problems of the European Society of Intensive Care Medicine Intensive Care Med 1996 22 707 710 10.1007/BF01709751 8844239

[B36] Iapichino G Radrizzani D Bertolini G Ferla L Pasetti G Pezzi A Porta F Miranda DR Daily classification of the level of care. A method to describe clinical course of illness, use of resources and quality of intensive care assistance Intensive Care Med 2001 27 131 136 10.1007/s001340000776 11280624

[B37] Pandharipande PP Sanders RD Girard TD McGrane S Thompson JL Shintani AK Herr DL Maze M Ely EW Effect of dexmedetomidine versus lorazepam on outcome in patients with sepsis: an a priori-designed analysis of the MENDS randomized controlled trial Crit Care 2010 14 R38 10.1186/cc8916 20233428 PMC2887145

[B38] Inouye SK Delirium in older persons N Engl J Med 2006 354 1157 1165 10.1056/NEJMra052321 16540616

[B39] Zeger SL Liang KY An overview of methods for the analysis of longitudinal data Stat Med 1992 11 1825 1839 10.1002/sim.4780111406 1480876

[B40] Mattia C Savoia G Paoletti F Piazza O Albanese D Amantea B Ambrosio F Belfiore B Berti M Bertini L Bruno F Carassiti M Celleno D Coluzzi F Consales G Costantini A Cuppini F De Gaudio RA Farnia A Finco G Gravino E Guberti A Laurenzi L Mangione S Marano M Mariconda G Martorano PP Mediati R Mercieri M Mondello E SIAARTI recommendations for analgo-sedation in intensive care unit Minerva Anestesiol 2006 72 769 805 17006417

